# Subunit pI Can Influence Protein Complex Dissociation Characteristics

**DOI:** 10.1007/s13361-019-02198-3

**Published:** 2019-05-10

**Authors:** Aneika C. Leney

**Affiliations:** School of Biosciences, University of Birmingham, Edgbaston, Birmingham, B15 2TT UK

**Keywords:** Native mass spectrometry, Protein complex dissociation, Isoelectric point, Collision-induced dissociation, Phycobiliproteins

## Abstract

Mass spectrometry is frequently used to determine protein complex topology. By combining in-solution and gas-phase dissociation measurements, information can be indirectly inferred about the original composition of the protein complex. Although the mechanisms behind gas-phase complex dissociation are becoming more established, protein complex dissociation is not always predictable. Here, we looked into the effect of the protein subunits pI on complex dissociation. We chose two structurally similar, hexameric protein complexes that consist of a ring of alternating alpha and beta subunits. For one complex, allophycocyanin, the alpha and beta subunits are structurally similar, almost identical in mass, but have distinct pIs. In contrast, the other complex, phycoerythrin, is structural similar to allophycocyanin, yet the subunits have identical pIs. As predicted based on the structural arrangement, dissociation of phycoerythrin resulted in the observation of both the alpha and beta monomeric subunits in the mass spectrometer. However, for allophycocyanin, the results differed dramatically, with only the alpha monomeric subunit being detected upon gas-phase dissociation. Together, the results highlighted the importance of considering the isoelectric points of individual subunits within a protein complex when using tandem mass spectrometry data to elucidate protein complex topology.

**Electronic supplementary material:**

The online version of this article (10.1007/s13361-019-02198-3) contains supplementary material, which is available to authorized users.

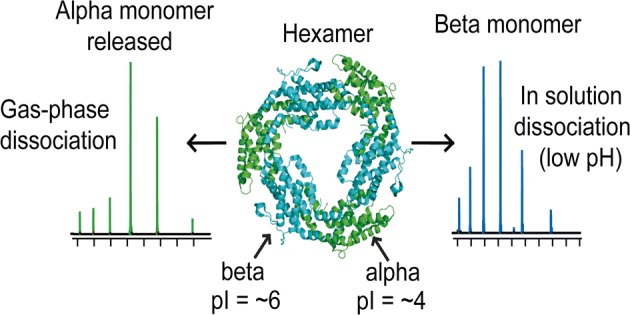

## Introduction

Thousands of proteins assemble into functional protein complexes to carry out their biological role. Deciphering how all these subunits interact within these complexes is a major aim for structural biologists. Thus, tools are continuously being developed to understand the subunit topology within protein complexes and dynamics that are displayed within these macromolecular assemblies. Mass spectrometry is being increasingly used in this area. Using native mass spectrometry techniques, protein complexes can be preserved into the gas phase for structural analysis [1–5]. Once in the gas phase, the protein complexes can then be sequentially dissociated; the order in which subunits are released and detected can be used to determine the architecture of the protein complex [6–8]. The most commonly used technique for gas-phase dissociation is collision-induced dissociation (CID). This typically results in the unfolding and release of a highly charged monomer and its corresponding “monomer-less” complex [9–12]. Despite this ejected monomer being unfolded and thus information lost as to its original structure, the nature of which monomer in a multicomponent complex that is ejected can still provide fruitful information. To highlight a few examples, mass spectrometry studies were used to generate a comprehensive interaction map for the eukaryotic initiation factor 3 that is composed of 13 distinct subunits [13]. In addition, tandem mass spectrometry experiments were able to aid localization of the stator subunits E and G in the A-ATPase membrane protein complex [14].

Another way to elucidate the topology of protein complexes using mass spectrometry is to stepwise dissociate the protein complex in solution by changing the ionic strength or pH, and then analyze its components in the mass spectrometer. This has been successfully done to characterize the scavenger decapping and nuclear cap-binding complexes extracted directly from *Saccharomyces cerevisiae* and the yeast exosome [15]. More recently, this technique has been applied to look at large viral capsids whose overall stoichiometry can be difficult to decipher based on the obtained *m*/*z* values alone [16, 17]. Moreover, by combining these measurements with ion mobility spectrometry, the assembly of protein complexes can also be monitored, for example, in monitoring the assembly intermediates of viruses [18] and deciphering building blocks of large amyloid-forming oligomers [19, 20]. Despite multiple examples of the use of tandem mass spectrometry in structural biology, the mechanism behind gas-phase protein complex dissociation is still not clear. Indeed, in-solution and gas-phase dissociation are not the same, and when differences are observed, it frequently leads us to question the biological relevance of the obtained data.

**Figure 1:**
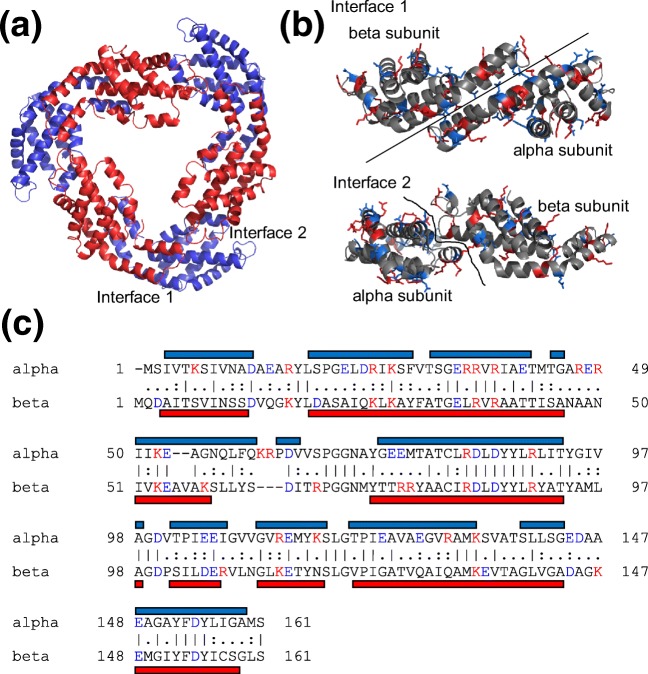
Structure and composition of allophycocyanin. (**a**) Allophycocyanin hexamer consisting of alpha (blue) and beta (red) subunits arranged alternatively in a ring structure (PDB: 1ALL). (**b**) Interaction interfaces. Interaction 1 represents the alpha-beta dimeric building block. (**c**) Sequence alignment of alpha and beta subunits highlighting the positively in red (K+R) and the negatively charged residue in blue (D+E). The helical regions are shown as a horizontal bar for both subunits

Here, we highlight an interesting example whereby the in-solution dissociation product ions at pH 2.5 observed for the protein complex allophycocyanin differ dramatically from its gas-phase dissociation product ions. Allophycocyanin is a blue-green colored, 107-kDa protein complex (Figure 1a) that resides in the phycobilisome, a large light harvesting complexes in red algae and cyanobacteria [21, 22]. In this MegaDa complex, allophycocyanin acts along with other structurally similar protein complexes (phycocyanin and phycoerythrin) to transmit light to the photosystem during photosynthesis [23]. Allophycocyanin is composed predominantly of alpha and beta subunits that assemble into a trimer of αβ dimers (Figure 1a, b). Upon gas-phase dissociation, only the alpha subunit of allophycocyanin hexamer was observed in contrast with phycoerythrin whereby both the alpha and beta subunits were released in a predictable manor. Upon comparison of protein sequences, we correlate these differences to the differential pI of the subunits present within the protein complexes. Together, the results highlight the importance of the pI of proteins when utilizing mass spectrometry–based detection methods for understanding the hierarchical assembly of protein complexes.

## Materials and Methods

### Chemicals and Reagents

All chemicals were purchased from Thermo Fisher Scientific. Allophycocyanin from *Arthrospira platensis* was purchased from Sigma-Aldrich and stored at 4 °C in the dark prior to use. B-phycoerythrin from *Porphorydium cruentum* was purchased from Thermo Fisher Scientific and stored at 4 °C in the dark prior to use.

### Native Mass Spectrometry

For native mass spectrometry, allophycocyanin and phycoerythrin (5–12 μM) were buffer exchanged into 100 mM ammonium acetate solution (pH ~ 7) by use of a 10,000 MWCO centrifugal filter (Amicon). Native mass spectrometry experiments were carried out on a Q-Exactive HF instrument (Thermo Fisher Scientific) coupled to a TriVersa NanoMate (Advion) to introduce the proteins into the mass spectrometer via nanoelectrospray ionization. A voltage of 1.8 kV was applied. The source temperature was set to 250 °C, in-source voltage 40, and higher-energy collisional dissociation (HCD) voltage 0. For allophycocyanin complex dissociation, the HCD collision energy was increased to 120. Mass spectra were acquired over a mass range of 1000–6000 *m*/*z*. For phycoerythrin, experiments were performed on an Orbitrap EMR instrument (Thermo Fisher Scientific) operated in positive ion mode. A nanoelectrospray source was used and a capillary voltage of 1.4 kV applied. The following parameters were used: source DC offset 25 V, injection flatapole 8 V, inter flatapole 7 V, bent flatapole 6 V, and transfer multiple 4 V. For complex dissociation, the HCD voltage was increased to 150. The instruments were calibrated with CsI, and the data analyzed using XCalibur (Thermo Fisher Scientific).

For gas-phase collision-induced dissociation measurements for allophycocyanin above 6000 *m*/*z*, a 7 Tesla solariX-XR FT-ICR mass spectrometer (Bruker) was used equipped with a TriVersa NanoMate (Advion). A voltage of 1.8 kV was applied in positive ion mode. The temperature of the dry gas was set to 150 °C. The radio-frequency (RF) amplitude of the ion-funnel was 150 V_pp_, and the applied voltage was 150 V for funnel 1 and 115 V for skimmer 1. The RF frequencies were set to 5 MHz, 1.4 MHz, and 1 MHz for the octapole, quadrupole, and transfer optics, respectively. Ions were accumulated for 50 ms. The time of flight was set to 1.8 ms. The vacuum pressures were ~ 3 mbar for the source region, ~ 8 × 10^−6^ mbar for the quadrupole region, and ~ 4 × 10^−9^ mbar for the UHV chamber. The in-source fragmentation was set to 100 V to aid desolvation. For complex dissociation, the most abundant charge states were selected (4600 *m*/*z* isolated with 800 isolation window) and dissociated using a collision energy from 5 to 35 V. Data were collected with 64 k of data points and 900 scans averaged. The spectra were calibrated externally with CsI.

In-solution, dissociation was performed by rapid dilution of the protein complexes into either 10% formic acid or 25% acetonitrile depending on the experiment performed. The solution was analyzed immediately on a Q-Exactive HF instrument using the settings described previously.

## Results and Discussion

**Figure 2:**
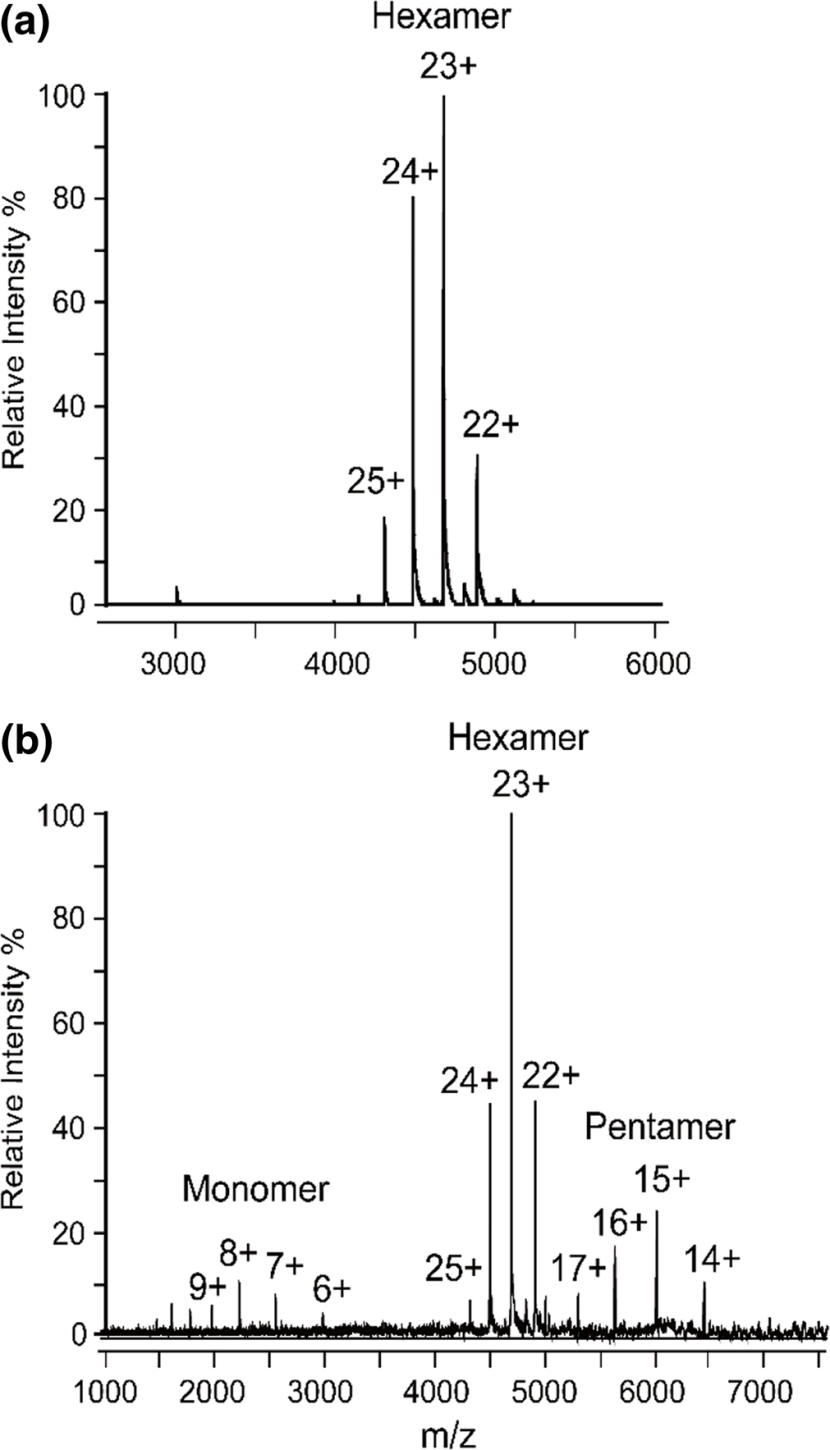
Allophycocyanin is a hexamer and displays typical collision-induced dissociation behavior. (**a**) Native mass spectrum of allophycocyanin. (**b**) Collision-induced dissociation of allophycocyanin showing release of monomer

**Figure 3:**
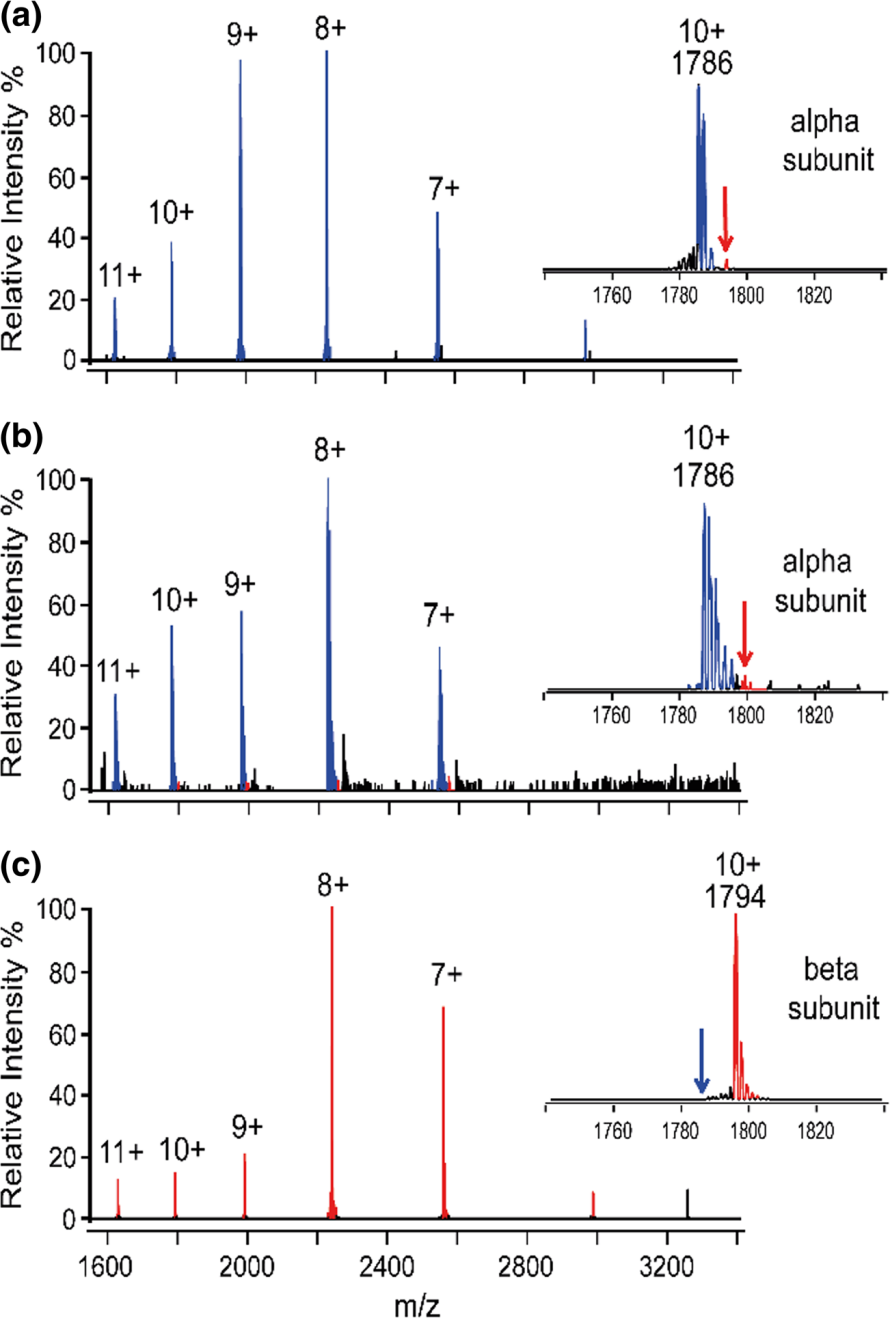
Allophycocyanin dissociates differentially in solution and in the gas phase. Mass spectrum in region 1600–3300 *m*/*z* of allophycocyanin upon (**a**) gas-phase dissociation and in-solution dissociation at (**b**) pH 7 and (**c**) pH 2.5. The inserts show a zoom in on the 10+ charge state highlighting which monomer was observed. The *m*/*z* positions of the alpha (blue) and beta (red) subunits are highlighted

The native mass spectrum of allophycocyanin is shown in Figure 2a. The major peaks correspond to a narrow charge distribution (21+ to 26+) for the allophycocyanin hexamer of mass 107,355 Da (Table 1). Upon CID, the hexamer dissociated into a monomer and its corresponding pentamer (Figure 2b). Considering the structural arrangement of the alpha and beta subunits in the allophycocyanin hexamer (Figure 1), the sequence and structural similarity (calculated RMSD 1.39 for structural alignment [24]) and their near identical mass (17,846 Da and 17,930 Da for α and β, respectively), we anticipated that there would be equal probability of an alpha or beta subunit being ejected from the complex upon CID. Thus, both the alpha and beta monomers would be detected and their corresponding α_3_β_2_ and α_2_β_3_ pentamers. Due to the small difference in mass (~ 80 Da on ~ 89,500 Da pentamer), the precise composition of the pentamer could not be resolved. However, interestingly, upon analysis of the ions corresponding to the monomers, only one major species was observed corresponded to the alpha subunit of allophycocyanin (Figures 2b and 3a) regardless of the collision energy used (Supplementary Figure 1). Moreover, very small peaks (< 2% alpha subunit peak intensity) were observed for the beta subunit showing these monomeric species if present can clearly be resolved.

**Table 1:** Expected and Observed Molecular Weights for Allophycocyanin and Phycoerythrin. The Alpha Subunit Mass for Allophycocyanin Is Reported with the Initial Methionine Residue Cleaved and with One Phycocyanobilin Molecule Attached. The Beta Subunit for Allophycocyanin Contains an Arg–Ala Mutation as Previously Reported [35] and with a Methylasparagine on Residue 71. The Expected Mass of the Hexamer Was Calculated Assuming the Presence of Both Alpha Proteoforms in a 50:50. One Hundred Percent of Sequence Coverage Was Achieved for Both of the Allophycocyanin Subunits by Digestion with Trypsin Followed by LC-MS/MS Analysis. The Beta Subunit for Phycoerythrin Contains a Methylasparagine on Residue 72. The pI Was Predicted in All Cases Assuming No Change in pI Occurred Upon Asparagine Methylation [36]

Protein	Expected MW (Da)	Observed MW (Da)	Mass deviation (%)	Predicted pI
Allophycocyanin alpha	17,846.3	17,845.6	0.002	4.89
Allophycocyanin alpha (+ Methylasparagine)	17,860.3	17,860.9	0.003	4.89
Allophycocyanin beta	17,929.5	17,928.6	0.005	6.25
Phycoerythrin alpha	18,976.4	18,976.6	0.001	5.42
Phycoerythrin beta	20,327.2	20,326.6	0.003	5.43
Allophycocyanin hexamer	107,348.4	107,354.9	0.006	–

Inspired by this intriguing observation, we next dissociated allophycocyanin in solution by the addition of acetonitrile while maintaining the pH at 7, and analyzed the dissociation products under native MS instrument conditions (Figure 3b). No peaks were visible corresponding to the intact hexamer proving complex dissociation had occurred. Yet again, only peaks corresponding to the alpha subunit of allophycocyanin were observed with baseline levels of the beta subunit ions detected (Figure 3b). Thus, we questioned the composition of the allophycocyanin hexamer. The intact mass, however, clearly corresponds to a mixture of three alpha and three beta subunits (Table 1), and upon protein digestion and LC-MS/MS analysis, both subunits were observed with 100% sequence coverage (data not shown). Moreover, upon in-solution dissociation of allophycocyanin by decreasing the pH to 2.5 (by the addition of formic acid), the beta subunit of allophycocyanin was clearly visible and dominated the mass spectrum (Figure 3c) proving that it is indeed present in solution and in the intact complex.

Since detection by mass spectrometry is highly dependent on charge, we hypothesized that, based on these observations, the protein subunits may be charged differentially when present in the hexameric complex altering their ability to be detected. Upon comparison of the amino acid sequences of the alpha and beta subunits, although structurally similar, their predicted pIs and thus their number of positive and negatively charged residues differ (Table 1). At pH 2.5, both the alpha (pI = 4.89) and beta subunit (pI = 6.25) will have a net positive charge in solution. Thus, one would expect, considering their identical size, that their ionization efficiencies are similar and the alpha and beta subunits would have equal abundances when detected in the mass spectrometer. However, only the beta subunit was detected (Figure 3c). Indeed, one likely explanation is that, upon decreasing the pH from 7 to 2.5, the alpha subunit crosses its pI in solution and precipitates preventing it from being detected. Interestingly, when the hexamer was dissociated in solution with the pH maintained at 7, the alpha subunit was predominantly observed (Figure 3b). The reasoning for this is currently unclear, though repeating these experiments in negative ion mode may shed some light into the mechanisms involved.

There are multiple factors contributing to the gas-phase dissociation behavior of protein complexes, for example, salt bridges, subunit flexibility and salt bridge formation [25, 26]. In addition, predicting precisely where the charge lies on a gas-phase protein complex is challenging. Previous studies have suggested that isomeric forms of homodimers may preexist in the gas phase prior to dissociation, whereby the charge on the complex is distributed asymmetrically across the two subunits [27]. However, other factors such as electron transfer occurring between protein complexes that contain metal ions [28], the removal of buffer anions altering the overall charge [29], and proton transfer between subunits occurring upon gas-phase activation [9, 11] can additionally alter the charged product ions observed. Moreover, when no disulphide bonds are present within the subunits, a model whereby subunit unfolding occurs prior to ejection of the leaving subunit best describes the asymmetric charge partitioning that occurs during CID [30]. In our case, since the subunits are structurally similar, are of similar mass, and both interfaces that need to be broken to release an alpha or beta subunit are identical, we hypothesize that another factor, the pI in addition to the overall number of positive and negative charges on the subunit, is having an effect on gas-phase complex dissociation. Moreover, the alpha subunit contains two more positively charged residues (18 Lys+Arg) compared with the beta subunit (16 Lys+Arg), and 7 more negatively charged residues (23 Asp+Glu) likely explaining it as a preferential release and thus the sole detection of the alpha subunit upon collision-induced dissociation.

**Figure 4:**
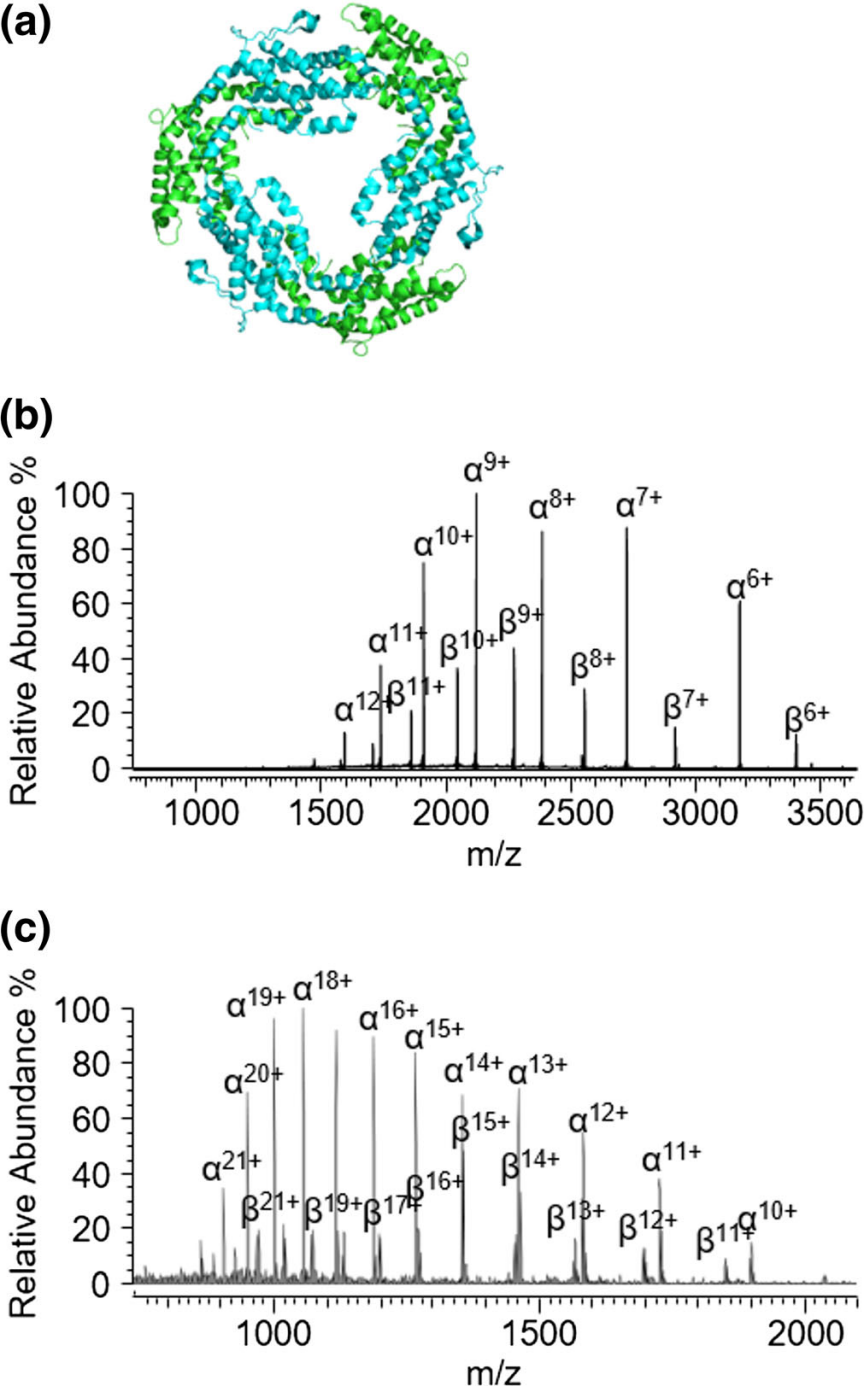
Both alpha and beta subunits are released upon phycoerythrin dissociation. (**a**) Phycoerythrin hexamer (PDB: 5aqd) [34] consisting of alpha (green) and beta (blue) subunits arranged in a similar manor to those in allophycocyanin. (**b**) Collision-induced dissociation of all ions corresponding to phycoerythrin showing released of both alpha and beta monomers. (**c**) In-solution dissociation of phycoerythrin at pH 2.5

To look into this further, we next investigated the gas-phase dissociation characteristics of a structurally similar protein complex, phycoerythrin, whose hexameric conformation also consists of alternating alpha and beta subunits arranged in a ring structure (Figure 4a). In contrast with allophycocyanin, however, the alpha and beta subunits of phycoerythrin are almost identical in pI (5.42 and 5.43 for the alpha and beta subunits, respectively). Thus, we hypothesized that if the pI difference between the alpha and beta subunits in allophycocyanin has an effect on its gas-phase dissociation behavior, then the gas-phase dissociation of the phycoerythrin hexamer would occur in a more “traditional” behavior and result in the observation of both alpha and beta ions in an approximately 1:1 ratio. At pH 7, phycoerythrin, unlike allophycocyanin, consists predominantly of an α_6_β_6_γ complex, whereby the gamma subunit plays a role in stabilizing the two hexameric α_3_β_3_ rings (Supplementary Figure 2) [31, 32]. Using native MS, it was shown that at pH 7, the α_3_β_3_ hexamer is present in equilibrium with its larger assembly [32] (Supplementary Figure 3). Upon all-ion fragmentation of the phycoerythrin, as predicted based on their similar pI values, both the alpha and beta subunits were observed at the low *m*/*z* regions (Figure 4b). Moreover, upon in-solution phycoerythrin complex dissociation by lowering the pH to pH 2.5, both the alpha and beta subunits were again clearly visible (Figure 4c) and consistent with their near identical pIs. The subunits abundances are slightly different to a 1:1 ratio as would be expected based on differences in ionization efficiencies.

Overall, the results highlight how the pI of protein subunits within a protein complex can alter its dissociation behavior. Although a single example is presented, we anticipate that these findings will be widespread among protein complexes. Moreover, without reengineering protein complexes to have distinct protein characteristics, finding structurally similar complexes whereby the subunits are of comparable size and structural arrangement to allow more of these types of investigations to be carried out is challenging.

## Conclusions

Mass spectrometry can provide a wealth of information about protein complex architecture. Yet, more experiments are still needed for us to fully understand this process. Indeed, several factors need to be taken into consideration for us to predict gas-phase dissociation behavior, for example, the subunit’s mass, conformation, structural flexibility and spatial arrangement. Here, we provide an example, whereby the pI of the protein subunits in solution enables one subunit to be preferentially released and detected over another structurally similar subunit with almost identical mass. Thus, charge plays an ever increasing role in gas-phase dissociation measurements. As such, care needs to be taken still when interpreting tandem mass spectrometry measurements on protein complexes, and where different subunits are observed between gas-phase dissociation and in-solution dissociation measurements, alternative dissociation methods such as surface-induced dissociation [33] are sought that could circumvent these issues. Finally, from our results, we suggest that if the finding is generic across a multitude of protein complexes, the predicted pI of proteins in solution could act as a useful parameter to predict gas-phase dissociation behavior of protein complexes.

## Electronic supplementary material

ESM 1 (DOCX 333 kb)
