# Supplementary material for: Subunit pI Can Influence Protein Complex Dissociation Characteristics
Source: J Am Soc Mass Spectrom. 2019 May 10;30(8):1389–95. doi: 10.1007/s13361-019-02198-3 (PMC6669198; doi:10.1007/s13361-019-02198-3)
Supplement: Supplementary file 1 — (DOCX 333 kb) [file 13361_2019_2198_MOESM1_ESM.docx]

**Supplementary Information**

**Subunit pI can influence protein complex dissociation characteristics**

Aneika C. Leney

School of Biosciences, University of Birmingham, Edgbaston, Birmingham, B15 2TT, UK.

B-phycoerythrin – P cruentum


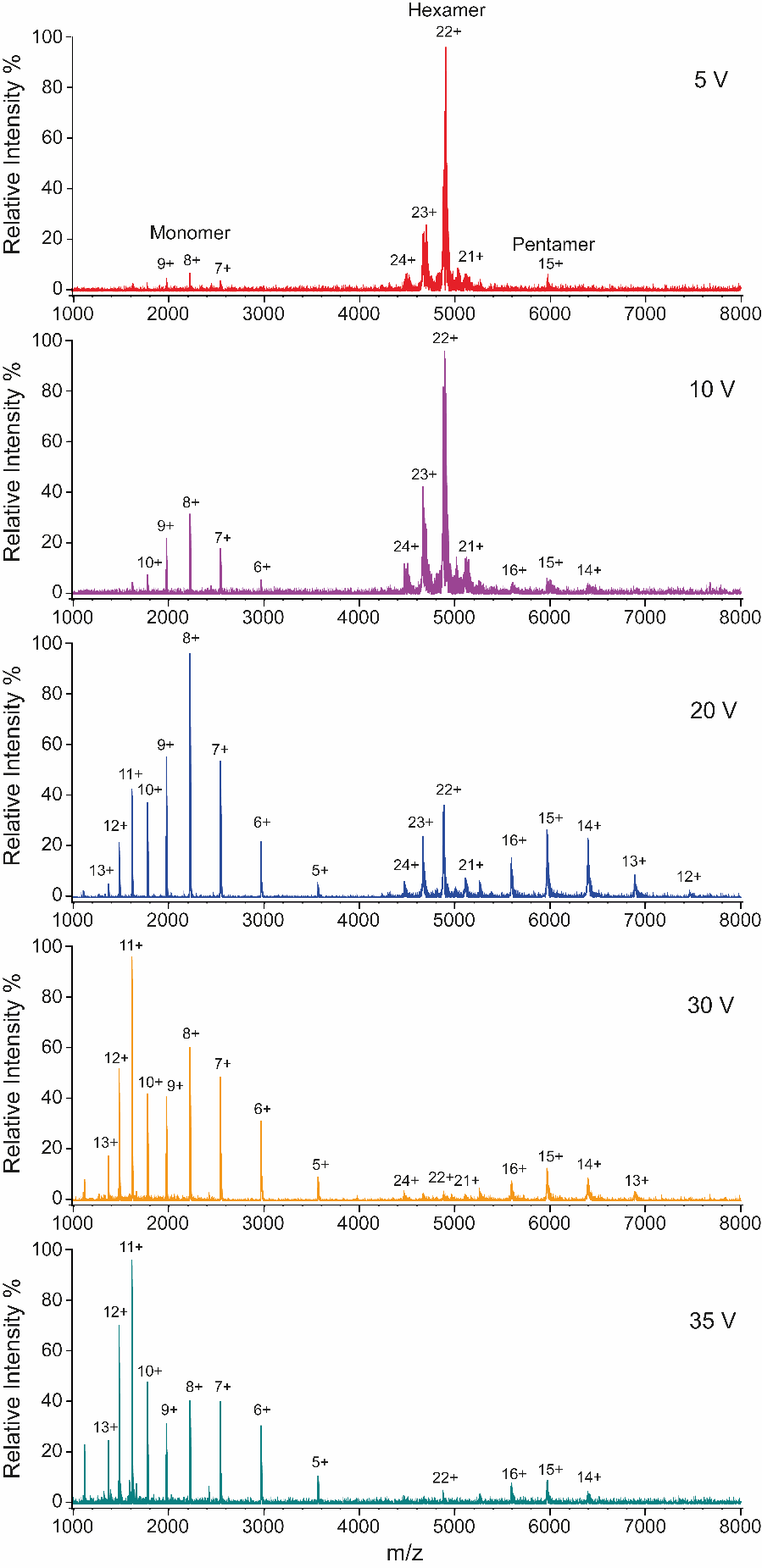


Figure S1. Collision induced dissociation of allophycocyanin hexamer at collision energies 5, 10, 20, 30 and 35 V. In all cases, only the monomer corresponding to the alpha subunit of allophycocyanin was detected.


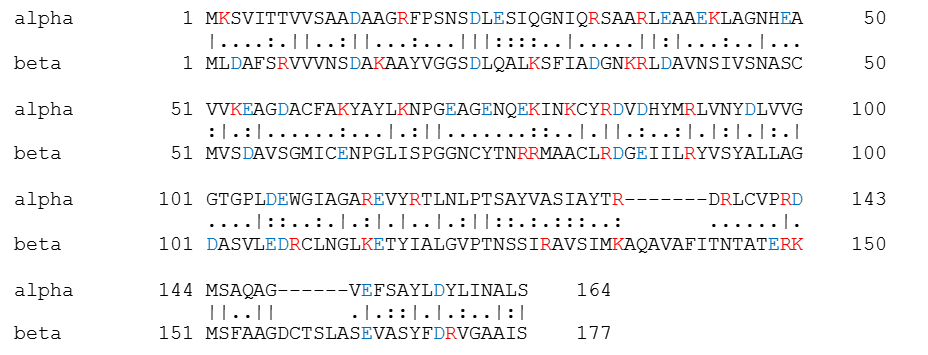


Figure S2. Sequence alignment of the alpha and beta subunits of B-phycoerythrin. The positive and negative charged residues are highlighted in red and blue, respectively.


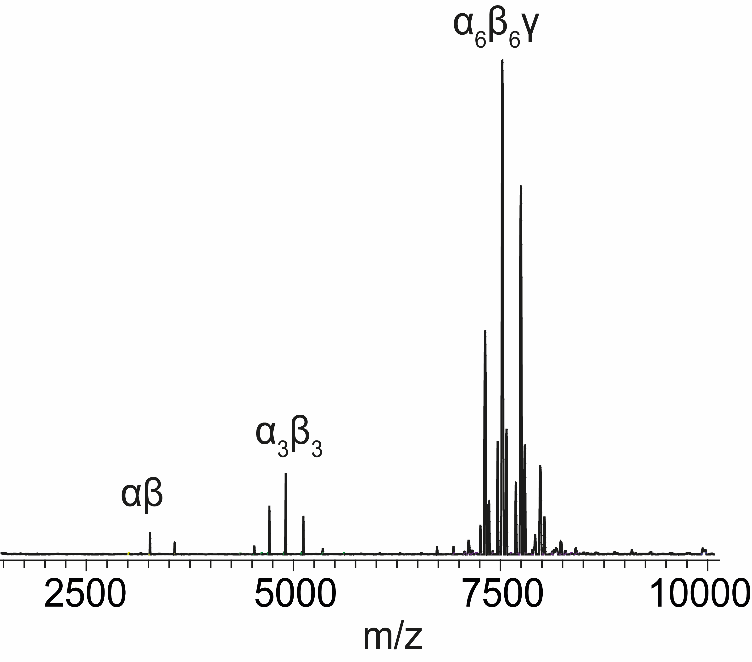


Figure S3. Native mass spectrum of B-phycoerythrin in 100 mM ammonium acetate pH ~7. The mass spectrum shows phycoerythrin in equilibrium between its larger assembly (α_6_β_6_γ) and its hexameric state (α_3_β_3_). It is important to note that with the instrument parameters used, the relative abundances of the species in the spectrum likely does not reflect their abundances in solution. Nevertheless, it is clear that both the hexameric and larger assembly are present in solution.
